# Impact of varying light intensities on morphology, phytochemistry, volatile compounds, and gene expression in *Thymus vulgaris* L.

**DOI:** 10.1371/journal.pone.0317840

**Published:** 2025-02-26

**Authors:** Zahra Hashemifar, Forough Sanjarian, Hassanali Naghdi Badi, Ali Mehrafarin

**Affiliations:** 1 Department of Plant Bio-products, Institute of Agricultural Biotechnology (IAB), National Institute of Genetic Engineering and Biotechnology (NIGEB), Tehran, Iran; 2 Department of Agronomy and Plant Breeding, Faculty of Agriculture, Shahed University, Tehran, Iran; 3 Medicinal Plants Research Center, Shahed University, Tehran, Iran; University of Kotli, PAKISTAN

## Abstract

Light is a crucial factor in plant growth and development. Plants exposed to light stress experience various effects on their growth. This research was conducted to investigate the effects of different light intensities on morpho-physiological traits, phytochemical compounds, and gene expression related to the biosynthesis of voletile in *Thymus vulgaris* L. The results demonstrated that light intensity (20, 50, 70 and 100%) had a significant impact on morpho-physiological characteristics, pigments content, antioxidant enzymes activities, as well as the content of MDA, H_2_O_2_, anthocyanin, thymol, carvacrol, phenols, flavonoids, essential oils, and monoterpenes. Moreover, the expression of the biosynthesis genes of monoterpene compounds was significantly influenced by light intensity. While an increase in light intensity led to higher leaf count (164.6%) and biomass (33.5%), it was accompanied by a decrease in leaf area, stem length, and internode length. The highest levels of chlorophyll a (4.92 mgg^-1^ FW) and b (1.75 mgg^-1^ FW), carotenoids (907.31 µ Mg^-1^FW), MDA (9.93 µ Mg^-1^FW), anthocyanin, SOD (29.62 Umg^ − 1^ Protein), thymol (41.2%), and carvacrol (4.46%) were observed at 70% treatment and decreased as light intensity increased. Also, H_2_O_2_, catalase and polyphenol oxidase activities, phenols, flavonoids, essential oils, and monoterpenes increased with higher light intensity, with the highest H_2_O_2_ concentration recorded at 100% (4.43 fold). Importantly, key genes involved in monoterpene biosynthesis, including *DXR*, *TPS*, *CYP71D178*, and *CYP71D179*, exhibited significantly enhanced expression under full light conditions compared to other light intensities. In conclusion, increased light intensity stimulated the elevation of oxidative indicators, antioxidant activity and enhancing the expression of genes involved in phytochemical compound biosynthesis and consequently leading to the accumulation of volatile compounds in *Thymus vulgaris* L. Future research will focus on investigating the combined effects of various abiotic stresses at the field level and extending the stress duration to evaluate potential additive effects.

## 1. Introduction

Light is a crucial factor in photosynthesis and the growth and development of plants. Plants are often exposed to different light conditions, some of which can be stressful. This light stress can impede photosynthesis, affect the antioxidant system, carbon and nitrogen fixation, and impact various growth characteristics in plants. In order to cope with light stress, plants adapt their cellular, biochemical, and molecular mechanisms [1,2]. Moreover, light and its associated parameters significantly influence the production of secondary metabolites in plants. These secondary metabolites are vital for enhancing the flavour, aroma, and resilience of plants in unfavourable environmental conditions, and they also contribute to plant defence against pathogens, pests, and diseases [3,4,5].

Reactive oxygen species (ROS) primarily arise as by-products during chloroplast, mitochondrial, and peroxisomal metabolism. The balance of ROS is typically maintained under normal conditions. However, disturbances such as high or low light conditions can disrupt this equilibrium, resulting in increased ROS production. To counteract stress, plants utilize an antioxidant system consisting of enzymatic antioxidants such as superoxide dismutase, peroxidases, glutathione reductase, and catalase, as well as non-enzymatic antioxidants like ascorbic acid, flavonoids, α-tocopherol, carotenoids, and others. This integrated system not only helps to mitigate ROS, but also regulates plant signaling, defense responses, and growth and development [6,7].

*Thymus vulgaris* L., commonly known as thyme and belonging to the Lamiaceae family, has been employed for centuries as both a flavouring agent and in medicinal contexts. *Thymus vulgaris* L. is recognized for its therapeutic properties, including its utility in treating colds, diabetes, infections, digestive disorders, and parasitic infestations. It also exhibits soothing, antibiotic, and antifungal attributes [8]. The pharmaceutical, antibacterial, and antioxidant characteristics of *Thymus vulgaris* L. are contingent upon its bioactive components, such as thymol, carvacrol, geraniol, and others [9,10]. A significant portion of these compounds belongs to the terpenoid class. Terpenoids are synthesized via the MEP (2-C-Methylerythritol 4-phosphate) and MVA (acetate mevalonate) pathways. In plastids, Geranylgeranyl pyrophosphate (GGPP) derives from the MEP pathway, generating 1-Deoxy-D-xylulose 5-phosphate (DXP), which is subsequently reduced to MEP by 1-deoxy-D-xylulose 5-phosphate reductoisomerase (DXR), utilizing Nicotinamide adenine dinucleotide phosphate (NADPH) as a cofactor. MEP functions as a precursor for geranyl diphosphate (GPP) [11]. Terpene synthase enzymes (TPSs) are responsible for the conversion of GPP into monoterpenes and sesquiterpenes [12]. Cytochrome P450 monooxygenases (CYPs) assume pivotal roles in plant defense, mediating the biosynthesis and modulation of hormones, biopolymers, defense compounds (including terpenoids and flavonoids), fatty acids, among other functions [13,14]. The formation of thymol and carvacrol involves the hydroxylation of γ-terpinene at the 3- or 6-position, catalyzed by CYPs from the 71D subfamily [12].

Şeker et al. (2023) conducted a shade treatment study on *Salvia officinalis* L. *Origanum vulgar* L. over a two-year period. The results indicated that chlorophyll levels increased with the increasing shade. The highest chlorophyll concentration was observed in plants subjected to 75% shade [15]. Similarly, Ilić et al. (2024) reported that the cellular antioxidant activity in *Ocimum basilicum* L. and *Thymus vulgaris* L. under shade treatment surpassed that of plants grown in non-shade conditions [16]. Furthermore, among *Lippia gracili* exposed to coloured shade treatments, blue light conditions resulted in enhanced growth, essential oil accumulation, and increased flavonoid and pigment content [17].

In order to gain a comprehensive understanding of the effects of light intensity on *Thymus vulgaris* L., this study evaluated the changes in morpho-physiological and phytochemical traits, as well as expression of genes related to the synthesis of secondary metabolites. In addition, the role and relationship between examined traits and the amount of essential oil and terpenoids synthesis were analysed. The findings of this study have revealed the effect of light intensity on both quantitative and qualitative traits of *Thymus vulgaris* L., as well as the role of gene expression related to the biosynthesis pathway of their secondary metabolites.

## 2. Materials and methods

### 2.1 Plant materials and experimental site

**Fig 1:**
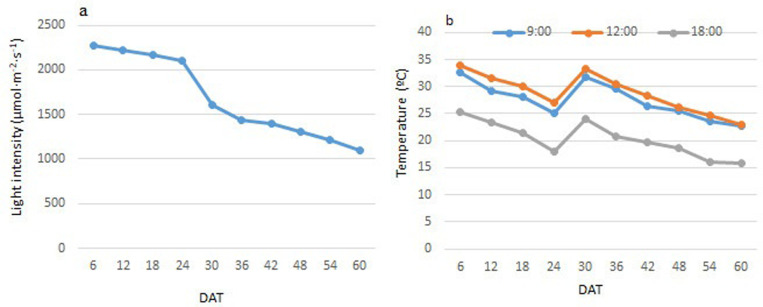
Environmental conditions during the growth period of *Thymus vulgaris* recorded on different days after treatments (DAT). (A) Light intensity (μmol m^-2^ s^-1^), (B) Temperatures at 9:00, 12:00, and 18:00 (ºC).

This experiment was conducted in the research greenhouse of the Institute of Medicinal Plants, ACECR, Karaj, Iran (56 ◦, 35′N and 50 ◦, 58′E, 1475 m above sea level). Rooted cuttings were planted in experimental pots. Then, irrigation and other field operations were carried out as needed. The plants planted in pots were initially grown in a greenhouse at a temperature of 25 °C with a humidity of 60% under the same conditions of the greenhouse for a period of six months before the light treatments commenced. Then, the plants were subjected to varying degrees of shading. Polyethylene shade nets of different thicknesses were used to achieve light intensity intensities of 20%, 50%, and 70%. A lux meter (INS DX-200, INS Enterprise Co., Ltd., Taiwan) was used to measure the light intensity under each shade. Three groups were exposed to direct sunlight as the control, representing 100% of natural sunlight intensity. The light intensity varied throughout the growth period, ranging from 1100 to 2270 μmol m^-2^ s^-1^, which is typical of full sunlight on a sunny day (Fig 1a). Daily records of the maximum and minimum temperatures under each shade were measured. Additionally, temperature readings under the various shades were documented at 9:00 AM, 12:00 PM, and 6:00 PM (Fig 1b).

### 2.2 Morphological parameters

The various morphological traits, including stem length (SL) (cm), stem diameter (SD)(mm), stem number (SN), number of leaves in the main stem (LN), leaf area (LA) (cm^2^), number of internodes (IN), and internode length (IL)(cm), were assessed. For leaf area measurement, standardized photos of the leaves were captured, and their areas were computed using IMAGE J 1.52v software. The entire aerial portions of the plants were removed from the soil surface and weighed for fresh weight. Subsequently, the shoots were dried under shade conditions at room temperature for a period of two weeks, following which their dry weights were recorded.

### 2.3 Photosynthetic pigments

The chlorophyll (Chl) and carotenoid (Cx + c) content was determined using the method developed by Lichtenthaler in 1987. A total of 200 mg of powdered plant leaves were mixed with 5 ml of 80% acetone. The tubes were centrifuged at 5000 rpm for 5 minutes, and the supernatant was collected. The concentrations of chlorophylls and carotenoids were measured at wavelengths of 470 nm, 645 nm, and 664 nm using a spectrophotometer (Specord 50, Analytik Jena, Germany). The amount of each pigment was calculated using the formula below:


$$Chla\left(mgg^{-1}\;FW\right)=\left(12.25A_{664}-2.79A_{645}\right)$$

$$Chlb\left(mgg^{-1}\;FW\right)=\left(21.51A_{645}-5.1A_{664}\right)$$

$$ChlTotal\left(mgg^{-1}\;FW\right)=Chla+Chlb$$

$$\text{Cx}+\text{c }\left({\text{mgg}}^{-1}\text{FW}\right)=\frac{\left(1000{\text{A}}_{470}-1.8\text{Chl a}-85.25\text{Chl b}\right)}{198}$$

[18].

### 2.4 Primary metabolites

The total sugar content (TSC) was determined calorimetrically using the anthrone method [19]. 200 mg shoot samples were ground in 2 ml 100 mM phosphate buffer solution and centrifuged at 4° C for 20 min at 10,000 rpm. The supernatant was used to measure total protein concentration and enzyme activity.

Protein content was measured using the Bradford method for protein dye-binding, with bovine serum albumin as the reference standard [20].

### 2.5 Oxidative indicators

The concentration of malondialdehyde (MDA) was measured to evaluate lipid peroxidation in the shoot samples. A total of 200 mg of shoot powder was added to a solution containing trichloroacetic acid (TCA) 20% (w/v) and thiobarbituric acid (TBA) 0.5% (w/v). The tubes were then heat-treated in a water bath at 95 °C for 30 minutes. After heating, the mixture was promptly cooled and centrifuged at 10,000 rpm for 10 minutes at 4 °C. The absorbance of each specific component was measured at a wavelength of 532 nm, while the absorbance of non-specific components was measured at 600 nm. The MDA concentration was calculated by subtracting the non-specific absorbance from the specific absorbance, using an attenuation value of 155 mM ^-1^ cm ^-1^ [21].

Another indicator that we measured was H_2_O_2_ content. Two hundred milligrams of plant powder were mixed with 2 ml of cold 0.1% (w/v) trichloroacetic acid. The mixture was then centrifuged at 12,000 rpm for 15 minutes at 4°C. Next, 0.5 ml of the supernatant was combined with 0.5 ml of 0.1 mM potassium phosphate buffer (pH 7) and 1 ml of potassium iodide (KI). The optical density (OD) of the reaction was measured at 390 nm, and the concentration of hydrogen peroxide in the plant tissue was determined using a standard curve [22].

### 2.6 Non-enzymatic antioxidants

Preparation of methanolic extract: Two grams of plant material were mixed with 15 mL of 70% methanol and subjected to maceration with continuous agitation for 24 hours. After incubation, the samples were centrifuged at 4000 rpm for 10 minutes. The resulting supernatants were collected and stored at -20 °C until further analysis.

#### 2.6.1 Total phenolic content (TPC).

To determine the total phenolic content, 180 µl of a methanolic extract was combined with 10 µl of Folin-Ciocalteu phenol reagent and 2400 µl of distilled water. The resulting mixture was allowed to stand at room temperature for 5 minutes. Subsequently, 500 µl of 20% sodium carbonate was added, and the solution was kept in complete darkness for 2 hours. The absorbance was measured at 740 nm using a spectrophotometer. The standard curve was created using gallic acid.

#### 2.6.2 Total flavonoids content (TFC).

A solution was prepared by combining 500 μL of methanolic extract, 1500 μL of methanol, 100 μL of 10% aluminum chloride, 100 μL of 1 M sodium acetate, and 2.8 mL of distilled water. The reaction solution was then kept in the dark for 30 minutes. Afterward, the absorbance was measured at a wavelength of 415 nm. Quercetin was used as a reference standard to construct the standard curve.

#### 2.6.3 Total anthocyanin content.

The methanolic extract mixed by HCL1% (99:1 ratio) and transferred into a 3 ml cell. The absorbance was measured in triplicate at a wavelength of 530 nm using a spectrophotometer. The sample concentration was calculated using the following formula, where A is the measured optical density (OD), with results expressed as milligrams per gram of sample.

### 2.7. Antioxidant enzymes

A sample of 200 mg of liquid nitrogen-frozen shoot samples were ground in 2 mL of phosphate buffer solution (100 mM) and centrifuged at 4 °C for 20 min at 10,000 rpm. The supernatant was tested for total protein concentration and enzymes activities.

#### 2.7.1 Superoxide dismutase (SOD) enzyme.

SOD activity was assessed using a reaction solution containing sodium phosphate buffer (50 mM), methionine (13 mM), NBT (75 μM), EDTA (0.200 mM), riboflavin (20 μM), and 180 μL of enzyme extract. The reaction solution was exposed to 15 V irradiated light at a distance of 1 cm for 20 minutes. Immediately after this exposure, the absorbance was measured at a wavelength of 560 nm. SOD activity was expressed as Umg^-1^ protein, reflecting the enzyme’s ability to inhibit the photochemical reduction of nitroblue tetrazolium (NBT). The SOD enzyme activity was calculated, with one unit of enzyme activity defined as the amount required to inhibit 50% of the photoreduction reaction.

#### 2.7.2 Catalase (CAT) enzyme.

A solution was prepared by combining 100 μL of leaf enzymatic extract, 100 μL of H_2_O_2_, and 2800 μL of phosphate buffer (50 mM, pH 7). The decrease optical density (OD) at 240 nm, due to the decomposition of H_2_O_2_, was examined. Measurements were taken at room temperature (24 °C) using a spectrophotometer at 10-second intervals over a duration of 3 minutes. As a control, H_2_O_2_ was replaced with an enzymatic extract in combination with the buffer solution.

#### 2.7.3 Peroxidase (POD) enzyme.

The reaction mixture was prepared with a total volume of 3 mL, consisting of 50 mM phosphate buffer (pH 7), 0.5% H_2_O_2_, 80 mM guaiacol, and 100 µ L of shoot enzyme extract. The absorbance of the reaction mixture was measured at a wavelength of 470 nm, with readings taken every 10 seconds for 3 minutes at 20 °C using a spectrophotometer. Each treatment was measured in triplicate. The buffer served as a blank, replacing the enzyme extract while retaining the other components.

#### 2.7.4 Polyphenol oxidase (PPO) enzyme.

In this test, the activity of the PPO enzyme is measured by the amount of pyrogallol converted to purpurgalin. The tubes were pre-warmed to 40°C in a water bath. Then, 2800 μL of phosphate buffer (50 mM, pH 7) was added to the tubes and allowed to equilibrate at 40°C. Next, 100 μL of enzyme extract was introduced. Finally, 0.2 mL of pyrogallol (20 mM) was added to the reaction mixture, and the enzyme activity was immediately assessed by measuring the decrease in optical density (OD) at 420 nm.

### 2.8 Essential oil analysis

#### 2.8.1. Isolation of the Essential Oil.

The aerial and underground parts of the plant were dried at room temperature for 14 days in a shaded room at 25°C. After drying, the plant materials were ground, and hydro-distillation was performed for 4 hours using a Clevenger-type apparatus.

#### 2.8.2. Gas Chromatography (GC) and Gas Chromatography-Mass Spectrometry (GC-MS).

GC analysis was conducted on an Agilent 6890 gas chromatograph equipped with a single injector and a flame ionization detector (FID). An apolar DB-5 capillary column was utilized with helium as the carrier gas at a flow rate of 0.8 mL/min. The oven temperature program was initiated at 50°C and held for 5 minutes, followed by a ramp of 15°C/min to 240°C and a final hold at 300°C for 3 minutes. The injector temperature was set at 290°C, and 1 μL of each sample was injected.

GC-MS analysis was performed using an Agilent 6890 gas chromatograph coupled with an Agilent 5973 mass selective detector. The mass spectrometer operated in electron impact mode (EIMS) with an electron energy of 70 eV, a scan range of 50–550 amu, and a scan rate of 3.99 scans/s. A fused silica HP-5ms capillary column (30 m ×  0.25 mm, 0.25 μm film thickness) was used for separation. Helium was used as the carrier gas at a flow rate of 0.8 mL/min. The injection temperature was set to 290°C. The oven temperature program began with a 5-minute hold at 50°C, followed by a ramp of 3°C/min to 240°C and a final hold at 300°C for 3 minutes.

#### 2.8.3. Identification of constituents.

The mass spectra and retention indices of the essential oil constituents were compared with reference standards in the computer library, and identification was based on published literature and standard compound mass spectra. GC peak identification was achieved through direct comparison of retention times (RT) and mass spectral data with standard compounds [23, 24].

### 2.9 Gene expression

The relative expression of the *DXR*, *TPS*, *CYP71D178*, and *CYP71D179* genes was evaluated through real-time PCR, with the *GAPDH* (Glyceraldehyde 3-phosphate dehydrogenase) gene used as the control. Total RNA was extracted from the aerial parts of all treatments and replications using the RNX-plus kit (EX6101, Sinaclon, Iran) according to the manufacturer’s instructions. RNase-free DNase I (Thermo Fisher Scientific Inc.) was utilized to eliminate any remaining DNA in the samples. Subsequently, cDNA synthesis was performed using the Add bio kit (Korea). Primer annealing temperatures were optimized through gradient PCR, and semi-quantitative RT-PCR was carried out using a 10 µl reaction volume. For qRT-PCR analysis, Amplicon master mix (ID 5000850-1250) and the Mic q-PCR cycler (Bio Molecular System) were employed. Gene expression analysis was conducted using REST 2009 software. The specifications of the primers can be found in S1 Table [25].

### 2.10 Statistical analysis

The experiments were conducted using a completely randomized block design with three replications. The data were normalized and analysed using SAS 9.4 software. Mean values were compared using Duncan’s multiple range test. Additionally, correlation tests and principal component analysis were performed using SPSS 16.0 software. Leaf area measurements were conducted using IMAGE J 1.52v software. GC-MS analyses were performed using Chemstation software.

## 3. Results and discussion

Solar radiation plays a vital role as an environmental factor in agricultural systems, being indispensable for promoting optimal crop growth, facilitating proper morpho-physiological development, and achieving maximum yields. Light serves as the primary energy source for plant activities and exerts a significant influence on numerous aspects of plant growth and development, including morphogenesis, photosynthesis, and metabolic processes [26].

### 3.1 Effects of light intensities on morphological traits

A thorough evaluation of morphological parameters, such as leaf numbers, internode length, internode number, stem number, stem diameter, stem length, and leaf area, revealed that the greatest values of internode length, stem length, stem count, and leaf area were observed at a light intensity of 20%. Additionally, the highest leaf numbers were achieved under full light conditions (100%). However, no significant differences were found in the number of internodes and stem diameter among different light intensities (Table 1).

**Table 1:** Different aspects of *Thymus vulgaris* L. affected by changes in light intensity.

Light intensity (%)	20	50	70	100	*p*
**Morphology/ metabolite**					
Leaf number	13^b^	19^b^	28.67^a^	34.33^a^	**
Internode length (mm)	15.2^a^	11.97^b^	11.6^b,c^	10.33^c^	**
Internodes number	35.67^a^	25^b^	32.67^a,b^	34.67^a^	ns
Stem number	26.67^a^	19^b^	25.67^a^	24.67^a^	*
Stem diameter (mm)	2.67^a,b^	2.33^b^	3.33^a^	3.33^a^	ns
Stem Length (cm)	36.5^a^	32.67^a^	32.17^a^	25.67^b^	*
Leaf area (cm^2^)	15.08^a^	11.39^b^	11.17^b^	9.86^b^	*
Sugar (mgg^-1^FW)	6.79^b^	5.62^b^	8.39^a^	9.27^a^	*
Protein (mgg^-1^FW)	167.78^a^	170.992^a^	176.243^a^	101.993^b^	***
Biomass (%)	35.73^c^	42.68^b^	43.17^b^	47.7^a^	*
**Photosynthetic pigments/ carotenoids**					
Total chlorophyll (mgg^-1^ FW)	6.046^b^	6.22^b^	6.68^a^	5.92^b^	*
Chlorophyll a (mgg^-1^ FW)	4.68^b^	4.86^a^	4.92^a^	4.29^c^	***
Chlorophyll b (mgg^-1^ FW)	1.37^b^	1.35^b^	1.75^a^	1.63^a^	*
Carotenoids (mgg^-1^ FW)	1.42^c^	1.52^b^	1.63^a^	1.62^a^	***
**Oxidative indicators/ non-enzymatic antioxidant**					
H_2_O_2_ (µMg^-1^FW)	71.83^c^	75.96^c^	93.56^b^	318.32^a^	***
MDA (µMg^-1^FW)	658.71^c^	497.85^d^	907.31^a^	857.85^b^	***
TPC (mgg^-1^ FW)	68.76^b^	56.30^d^	63.96^c^	77.69^a^	***
TFC (mgg^-1^ FW)	0.38^c^	0.34^d^	0.72^b^	1.14^a^	***
Anthocyanin (µMg^-1^FW)	6.63^c^	9.42^a,b^	9.93^a^	8.47^b^	**

***p ≤  0.001, **p ≤ 0.01, * *p* ≤ 0.05, ^ns^non-significant. Values followed by the same letter are not significantly different according to the Duncan test (*p* ≤  0.05).

*Thymus vulgaris* L. demonstrated longer internodes and main stems under shaded conditions, whereas increased light intensity resulted in shorter internodes and stems. The reduction in internode length and stem height at higher light intensity can be attributed to decreased apical dominance caused by a decrease in auxin levels at the stem apex [27]. Similar findings have been reported for other plant species, such as Sweet basil, which exhibited taller growth under shade conditions compared to open environments [28]. Interestingly, the leaf surface index on the main stem increased with rising light intensity, contributing to enhanced photosynthesis (Table 1). In contrast, Fernandes et al. reported a decrease in leaf area with increasing light radiation in *Ocimum gratissimum* [29].

### 3.2 Photosynthetic and growth characteristics change with varying light intensity

Changes in light intensity had a significant impact on the levels of chlorophyll a (Chl a), chlorophyll b (Chl b), and carotenoids (CAR). As the light intensity increased, the levels of these pigments also increased. The maximum amounts of Chl a, Chl b, and total chlorophyll content (Chl T) were observed at a light intensity of 70%. However, exposure to full light conditions resulted in a noticeable decrease in Chl a, and Chl T content by 12.8% and 11.38% respectively, although this reduction in Chl b was not statistically significant. The highest carotenoid content was observed under the 70% light treatment, showing an approximate increase of 15% (Table 1).

The intensity and quality of light also impact the biosynthesis of chlorophyll in plants. High light intensity can result in a decrease in chlorophyll accumulation and its precursors [30,31]. In this study, chlorophyll and carotenoid contents increased with higher light intensity, reaching their peaks at 70% light intensity (Table 1). The specific light intensity at which these pigments peak can vary among plant species, with *Ocimum tenuiflorum* L. reaching this point at 50% light intensity for chlorophyll and exhibiting higher carotenoid content under full sunlight [32]. In contrast, Lettuce showed lower carotenoid levels and higher chlorophyll content under shaded conditions [33].

The assessment of plant shoot biomass, expressed as a percentage of dry weight to fresh weight (%DW/FW), and revealed that the highest biomass was associated with the 100% light intensity (47.7%) (Table 1). The analysis of total sugar content (TSC) showed a significant increase with increasing light intensity, with the highest average TSC value recorded under 100% light intensity conditions (9.27 mgg^-1^FW). The measurement of protein content in *Thymus vulgaris* L. extracts, as determined by the Bradford test, exhibited the lowest protein content under the highest light intensity. No significant differences were observed among the 20%, 50%, and 70% light intensities (Table 1).

The highest biomass index was observed at 100% light intensity, which is consistent with the findings of Tabbert et al. (2021) that LED supplemental light increased biomass compared to natural light alone in *Thymus vulgaris* [34]. Additionally, the treatment with 100% light intensity was associated with the highest total TSC (Table 1). Tang et al. (2022) observed that light treatments significantly influenced soluble sugar, sucrose, and starch levels in *Medicago sativa*, with the highest levels occurring under high-light conditions [35]. Similarly, Castrillo et al. (2005) found that sugar content increased in plants exposed to full sunlight in various Mint species [36]. In contrast to biomass and TSC, protein content decreased under full sunlight conditions (Table 1). Although chlorophyll content per gram unit decreased under full light, the increase in the number of leaves and the leaf surface index in the main stem compensated for this deficiency, resulting in the plant having the maximum amount of sugar and biomass.

### 3.3 Effects of light intensities on oxidative indicators, and enzymatic and non-enzymatic antioxidants

Evaluation of H_2_O_2_ and malondialdehyde (MDA) content as indicators of oxidative stress revealed a noteworthy elevation in H_2_O_2_ content in plant tissues as light intensity increased, peaking under full light conditions. The accumulation of H_2_O_2_ at 100% light intensity surpassed that at 20% light intensity by more than fourfold. Conversely, the concentration of MDA, serving as an indicator of cell membrane degradation, exhibited the highest levels at 70% light intensity and the lowest at 50% intensity (Table 1).

**Fig 2:**
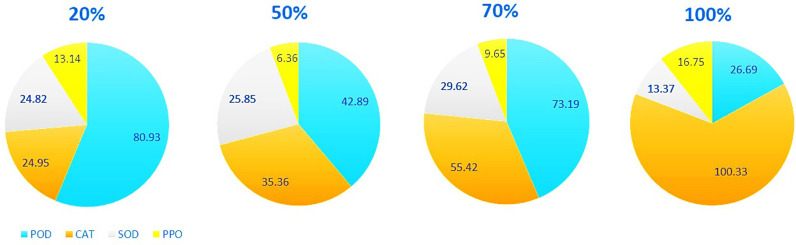
Alterations in the activity of antioxidant enzymes (U mg ^ − ^^1^ Protein) of *T. vulgaris* in response to different light intensities (20%, 50%, 70% and 100%).

The activity of four antioxidant enzymes was examined in *Thymus vulgaris* L. These enzymes exhibited varied responses to increasing light intensity. The CAT and PPO activities were highest under full light conditions, showing increases of 4.02 and 1.27 times, respectively, compared to the 20% light intensity. The POD displayed its peak activity at 20% light intensity, but its activity decreased as the light intensity increased, resulting in a 3.03-fold decrease under full light conditions. The highest SOD activity was recorded at 70% light intensity, subsequently decreasing by 2.22 times and reaching its lowest point under full light conditions (Fig 2). The TFC and TPC as non-enzymatic antioxidants were highest under full light conditions (1.14. mgg^-1^ FW and 77.69 mgg^-1^ FW, respectively), while the anthocyanin content was highest at 70% light intensity (9.93 µ Mg^-1^ FW) (Table 1).

Superoxide dismutase (SOD) is the primary defence mechanism against reactive oxygen species (ROS) within cells. It counteracts the formation of O_2_^-^, which occurs in various locations containing electron transport chains, including mitochondria, chloroplasts, peroxisomes, and more [37,38]. In this study, SOD enzyme activity demonstrated an additive response up to 70% light intensity, declining at higher light intensities. This trend was also observed for peroxidase (POD) enzyme activity, with both enzymes displaying reduced effectiveness at light intensities exceeding 70%. Under 100% light intensity, catalase (CAT) becomes the primary antioxidant enzyme, facilitating the conversion of H_2_O_2_ into water and oxygen in response to environmental stress. CAT is primarily located in sites of H_2_O_2_ production within plant cells, such as peroxisomes, mitochondria, cytosol, and chloroplasts. CAT deficiency can lead to plant anomalies, including chlorosis, head sterility, and increased sensitivity to normal photorespiratory conditions [39]. In this research, as light intensity increased, the amount of hydrogen peroxide in the aerial parts of the plant also increased (Table 1, Fig 2). In the correlation analysis of antioxidant enzymes activities, CAT enzyme activity exhibited the highest correlation with the amount of H_2_O_2_ (0.93) (Fig 5). Similarly, CAT activity corresponds to the amount of H_2_O_2_ in two Salvia species [40]. An increase in H_2_O_2_ and malondialdehyde (MDA) production has been associated with the up-regulation of secondary metabolite production under low light conditions in *Orthosiphon stamineus* seedlings [41]. Furthermore, shaded medicinal herbs like *Thymus vulgaris* L., *Origanum marjorana* L, and *Origanum vulgar* L. demonstrated higher antioxidant activity compared to unshaded control plants [42].

**Fig 3:**
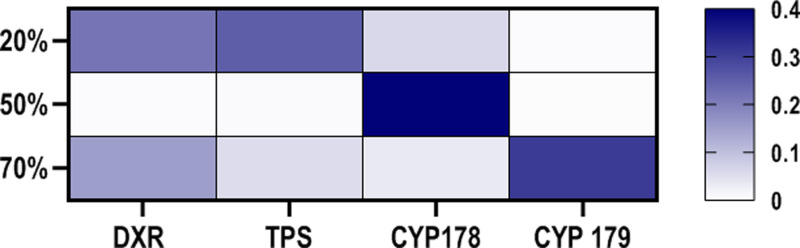
Heat map diagram illustrating the expression levels of genes involved in the thymol biosynthetic pathway in response to different light intensities in *T. vulgaris.* Error bars indicate the standard deviation.

**Fig 4:**
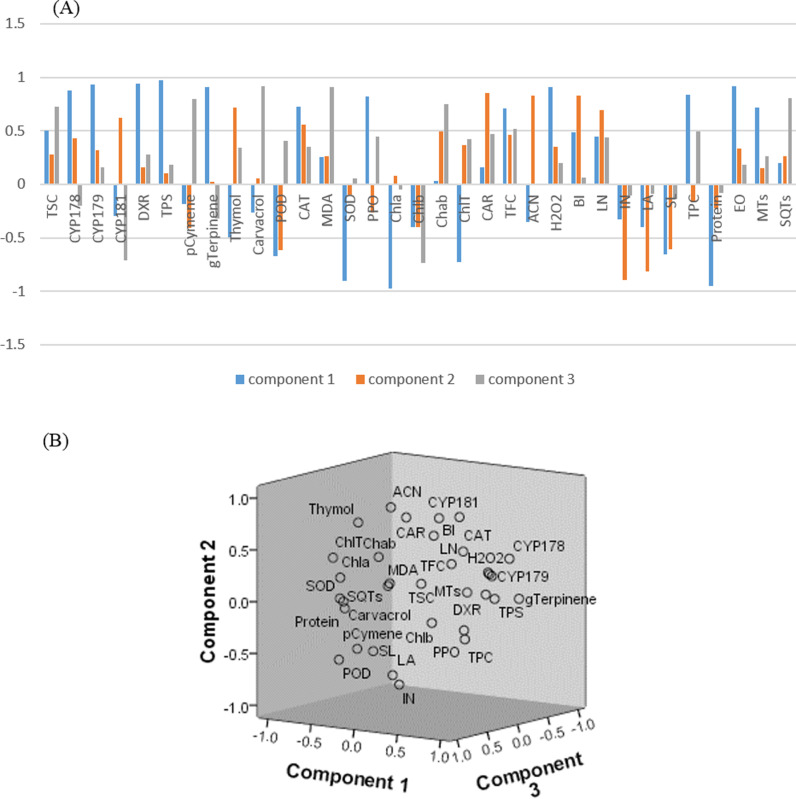
Principal Component Analysis (PCA) of studied traits in *Thymus vulgaris.* (A): Loading Plot, (B): Component Plot. TSC: Total sugar content, CYP178:*CYP71D178*, CYP179: *CYP71D179*, pCymen: P-Cymen, gTerpinene: γ-terpinene, POD: Peroxidase, CAT: Catalase, MDA: Malondialdehyde, SOD: super oxiddismotase, PPO: Polyphenol oxidase, Chla: Chlorophyll a, Chlb: Chlorophyll b, CAR: Carotenoids, TFC: Total flavonoid content, ACN: Anthocyanin, BI: Biomass index, LN: Leaf number, IN: Internode number, LA: Leaf area, SM: Stem length, TPC: Total phenol content, EO: Essential oil, MTs: Monoterpenes, SQTs: Sesquiterpenes.

**Fig 5:**
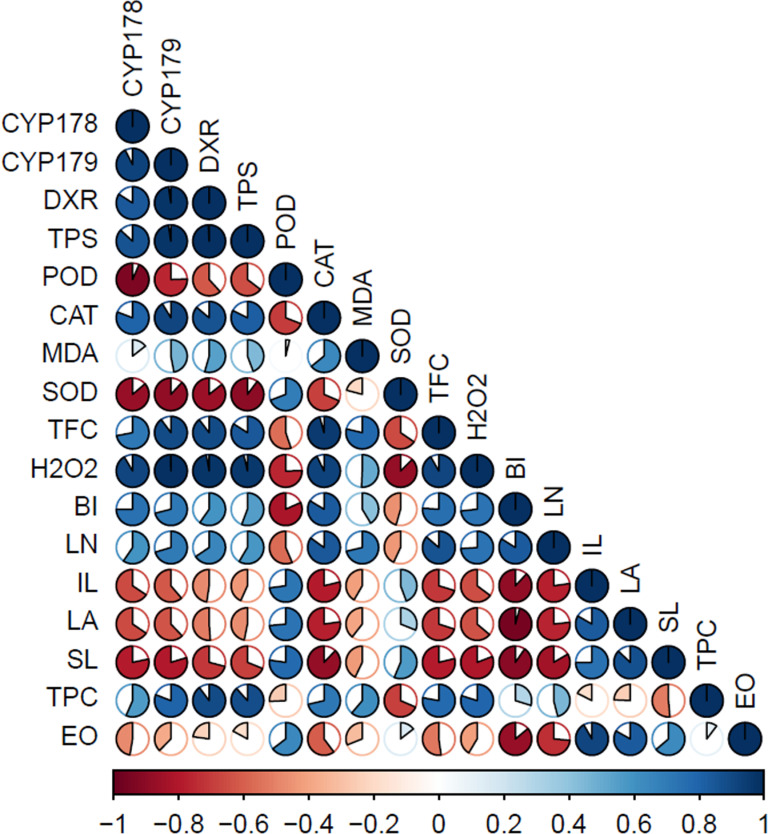
Pearson correlation between different traits in the *T. vulgaris.* CYP178: *CYP71D178*, CYP179: *CYP71D179*, EO: essential oil, BI: biomass index, LN: leaf number, IN: internode length, LA: leaf area, SL: stem number, CAT: catalase, POD: polyphenoloxidase, SOD: super oxiddismotase, MDA: malondialdehyde, TPC: total phenol content, TFC: total flavonoid content.

Flavonoids and phenolic acids have important functions as reducing agents, scavengers of free radicals, and quenchers of singlet oxygen formation. They play a crucial role in safeguarding biological systems against oxidative damage to macromolecules such as carbohydrates, proteins, lipids, and DNA [43]. These compounds are synthesized in response to various environmental factors, including exposure to light, and are vital for the defense mechanisms of injured plants [44]. In the presence of high light intensity resulting in increased levels of reactive oxygen species (ROS), *Thymus vulgaris* L. plants exhibited an elevated production of phenols and flavonoids (Table 1).

### 3.4 Effects of light intensities on Essential oil content and composition

The results indicated a significant increase in the content of the essential oil (EO) when exposed to full light intensity. Under 100% light intensity, the EO content was nearly ten times higher compared to the conditions with 20% light intensity. However, there were no statistically significant differences in EO content among the 20%, 50%, and 70% treatments, with mean values of 0.037%, 0.04% and 0.067%, respectively. Gas chromatography-mass spectrometry (GC-MS) analysis identified 26 compounds in the essential oil of plants control of *Thymus vulgaris* L., as specified in S2 Table. The major compounds included thymol, p-cymene, γ-terpinene, and carvacrol, constituting 39.24%, 22.1%, 10.59%, and 3.76% respectively, under full light conditions. Thymol and carvacrol showed their highest levels under 70% light intensity, with increases of 6.24% and 17.68% respectively, compared to 20% light intensity. γ-terpinene exhibited a 31.39% increase when exposed to full light compared to 20% light intensity, while p-cymene displayed a contrasting trend with a decrease of 10.78% under full light (S2 Table).

The production of essential oils is closely tied to the metabolic capacity of plants under optimal conditions of light intensity and quality, and this capacity varies significantly across different species [45]. Generally, higher levels of irradiance, increased rates of photosynthesis, and greater biomass accumulation are associated with essential oil production [46,47]. However, for shade-loving or sciophytic plants, higher levels of irradiance can trigger photo-inhibitory processes that result in reduced essential oil yields. Conversely, species adapted to high-light environments may experience a decrease in essential oil content when shaded, due to reduced rates of photosynthesis and biomass production [48]. For example, *Tetradenia riparia* exhibited higher essential oil content in 30% shade compared to full light conditions [49]. In contrast, *Thymus vulgaris* L. and *Origanum vulgar* L. showed higher essential oil content under full light conditions than in shaded environments [42,50,51].

*Thymus vulgaris* L. serves as a significant natural source of thymol, carvacrol, and some other valuable compounds. The composition of these components may vary depending on factors such as species, growth conditions, plant organ, developmental step, genotype, extraction process and other variables [52, 53]. The main compounds found in the essential oil of *T. vulgaris* were thymol, p-cymene, γ-terpinene, and carvacrol, which accounted for 39.24%, 22.1%, 10.59%, and 3.76% respectively, under full light conditions. Several species of Thymus, including *Thymus vulgaris* L., are known for their high antioxidant activity due to their rich content of carvacrol and thymol [54]. The positioning of the phenolic group in thymol, as opposed to carvacrol, gives it superior stability and reactivity as an antioxidant [42,54].

### 3.5 Impact of light intensities on the expression of terpenoid pathway genes

The impact of different light intensities on the expression of genes associated with thymol production, specifically those involved in the MEP pathway (*DXR*, *TPS* and thymol synthases *CYP71D178*, *CYP71D179*), was examined. The analysis of the relative expression levels of all four genes revealed a significant increase in gene expression level under higher light intensities. In particular, the relative expression level of the *CYP71D179* gene increased by 166.67-fold when the light intensity increased from 20% to 100%. Similarly, noteworthy increases in expression levels were observed for the *CYP71D178, DXR*, and *TPS* genes, with fold changes of 16.39, 4.63, and 4.33, respectively (Fig 3).

The *DXR* gene, which initiates the monoterpene synthesis pathway, showed the highest expression under full light conditions and demonstrated a strong correlation (0.99) with the *TPS* gene. These two genes collectively played a significant role in enhancing the level of γ-terpinene in the plant under full light conditions (Figs 3, 5; S2 Table).

In this investigation, the focus was on the elevated expression levels of the *CYP71D178* and *CYP71D179* genes, which encode enzymes that are essential for the biosynthesis of thymol and carvacrol, respectively. The study found that the expression of *CYP71D178* and *CYP71D179* significantly increased under high light intensity conditions. It was anticipated that this up-regulation of *CYP71D178* and *CYP71D179* expression in full light conditions would result in higher percentages of thymol and carvacrol in the essential oil. However, analysis of the essential oil revealed that the percentage of these two compounds was actually higher at 70% light intensity compared to 100% light intensity (S2 Table). It is possible that the higher light intensity led to an increased expression of *CYP736A300* and *CYP76S40*, enzymes that convert some thymol and carvacrol into thymohydroquinone and thymoquinone [12]. Furthermore, the emissions of thymol and carvacrol may increase at higher light intensities, as demonstrated in a study by Gouinguene and Turlings, which observed the emission of volatile compounds by young corn plants under high light intensities [55].

### 3.6 Principal component analysis (PCA) and correlation analysis

Principal Component Analysis (PCA) was performed to analyze the 32 traits studied in *Thymus vulgaris* L. The first two components accounted for more than 99% of the observed variation (86.96% and 12.33%, respectively) (Fig 4). Furthermore, a Pearson correlation analysis revealed that the essential oil (EO) exhibited a significant positive correlation of more than 0.9% with MEP pathway genes, TFC, H_2_O_2_, and CAT activity levels. Conversely, the biomass index demonstrated a significant negative correlation of more than 0.9% with SL and LA (Fig 5).

Principal Component Analysis (PCA) was performed to assess the influence of light intensity on multiple traits in *T. vulgaris*. The first component showed a strong relation with TSC, MDA, and CAT enzyme, accounting for 86.95% of the variance. The second component was linked to the expression of MEP pathway genes, γ-terpinene, and antioxidant enzymes SOD and POD, explaining 12.33% of the variance. Notably, MDA and TSC demonstrated the highest sensitivity to light variations (Fig 4).

## 4. Conclusion

Light intensity significantly affects various aspects of *Thymus vulgaris* L., including morphology, photosynthetic pigments content, antioxidant enzymes activities, antioxidant indices, essential oil quantity and composition, and the expression of related genes. At the gene expression level, 100% light intensity can increase the expression of terpenoid synthesis pathway (MEP) genes; however, at the metabolite and pigment levels, the results differ. High light intensity degrades pigments while increasing some antioxidants and decreasing others. In greenhouses, it is recommended to maintain light intensity above 1000 μmol m^-2^ s^-1^ during the final growth period to enhance the productivity of this medicinal plant. This study also provides valuable insights into the complex relationship between light intensity and various physiological and biochemical responses in *Thymus vulgaris* L. However, future investigations are recommended to explore these interactions under field conditions, where plants face various stresses. Such studies could address the potential variations in plant responses due to environmental complexities and evaluate how prolonged exposure to varying light intensities influences secondary metabolite production and stress tolerance mechanisms.

## Supporting information

S1 Table Characteristics of primers used for genes expression of methylerythritol phosphate (MEP) pathway in *Thymus vulgaris* L. (DOCX)

S2 Table The means of compounds of essential oil and its changes according to different levels of light intensity in *Thymus vulgaris* L. (DOCX)
