# Supplementary material for: Impact of varying light intensities on morphology, phytochemistry, volatile compounds, and gene expression in Thymus vulgaris L
Source: PLoS One. 2025 Feb 26;20(2):e0317840. doi: 10.1371/journal.pone.0317840 (PMC11864514; doi:10.1371/journal.pone.0317840)
Supplement: S1 Table — (DOCX) [file pone.0317840.s001.docx]

| **S1** **Table** Characteristics of primers used for genes expression of methylerythritol phosphate (MEP) pathway in *Thymus vulgaris* L. | | | |
| --- | --- | --- | --- |
| Genes |  | primers sequence (5ʹ-3ʹ) | Optimized annealing temperature (ºC) |
| *DXR* | Forward  Reverse | GAGGTGCTTTCAGGGATTTG  AGATTGGGGATGGATAACGAT | 54 |
|  |  |  |  |
| *TPS* | Forward  Reverse | GGCAAAATGGTACTACGCCG  AGTCGAGTTCGGGAGAGTGA | 59 |
|  |  |  |  |
| *CYP771D178* | Forward  Reverse | CAAGGAATGACTGCTGCTGAC  TTGGATTGTGGATTGTTGGAACC | 58 |
|  |  |  |  |
| *CYP771D179* | Forward  Reverse | CGTGGCTTCTCAACCTTCTC  CGCTCTTCTTCACCCTATGC | 58 |
|  |  |  |  |
|  |  |  |  |
| *GAPDH* | Forward | TCACTGACAAGGACAAGGCTG | 60 |
|  | Reverse | CTGGCTTCGCAAGTCTAACAG |  |
