# Supplementary material for: Impact of varying light intensities on morphology, phytochemistry, volatile compounds, and gene expression in Thymus vulgaris L
Source: PLoS One. 2025 Feb 26;20(2):e0317840. doi: 10.1371/journal.pone.0317840 (PMC11864514; doi:10.1371/journal.pone.0317840)
Supplement: S2 Table — (DOCX) [file pone.0317840.s002.docx]

| **S2 Table.** The means of compounds of essential oil and its changes according to different levels of light intensity in *Thymus vulgaris* L. | | | | | |
| --- | --- | --- | --- | --- | --- |
|  | 20% | 50% | 70% | 100% | p |
| α-Thujene | 0.67 | 0.53 | 0.32 | 0.66 | *** |
| α-Pinene | 1.19 | 1.09 | 0.6 | 1.08 | *** |
| Camphene | 1.1 | 1.31 | 0.63 | 1.04 | *** |
| β-Pinene | 0.25 | 0.23 | 0.15 | 0.3 | *** |
| Myrcene | 1.47 | 1.75 | 0.72 | 1.05 | *** |
| 3-Octanol | 0.26 | 0.61 | 0.19 | 0.26 | *** |
| α-Terpinene | 1.25 | 1.16 | 0.85 | 1.31 | *** |
| p-Cymene | 24.77 | 20.41 | 24.49 | 22.1 | * |
| Limonene | 0.74 | 0.67 | 0.63 | 0.48 | *** |
| Eucalyptol | 0.34 | 0.53 | 0.82 | 2.34 | *** |
| γ-Terpinene | 8.06 | 8.17 | 5.85 | 10.59 | *** |
| Linalool | 3.15 | 3.51 | 3.2 | 2.23 | *** |
| Camphor | 0.72 | 1.54 | 0.4 | 1 | *** |
| Borneol | 2.55 | 3.11 | 3.05 | 2.74 | *** |
| Terpinen-4-ol | 0.11 | 0.15 | 0.17 | 0.61 | *** |
| α-Terpineol | 0.19 | 0.24 | 0.25 | 0.45 | *** |
| Thymol methyl ether | 0.32 | 0.59 | 0.38 | 0.44 | ** |
| Thymol | 38.63 | 39.94 | 41.2 | 39.24 | *** |
| Carvacrol | 3.79 | 3.24 | 4.46 | 3.76 | *** |
| Caryophyllene | 0.13 | 1.99 | 1.89 | 2.25 | *** |
| Aromandendrene | 2.09 | 0.12 | 0.16 | 0.27 | *** |
| Bicyclogermacrene | 0.15 | 0.23 | 0.16 | 0.26 | *** |
| γ-Cadinene | 0.23 | 0.34 | 0.32 | 0.16 | *** |
| δ-Cadinene | 0.24 | 0.23 | 0.28 | 0.23 | ns |
| β-Spathulenol | 0.35 | 0.37 | 0.37 | 0.73 | *** |
| β-Caryophyllene oxide | 1.19 | 1.07 | 1.89 | 0.98 | *** |
| Mono-terpens | 87.3 | 86.1 | 87.9 | 90.98 | ** |
| Sesquiterpenes | 4.43 | 3.4 | 5.07 | 4.88 | * |
| Essential oil (%) | 0.037b | 0.04b | 0.067b | 0.49a | *** |

*** p ≤ 0.001, **p≤0.01, * p≤0.05, ns non-significant.
